# Deficiency of Lipin2 Results in Enhanced NF-κB Signaling and Osteoclast Formation in RAW-D Murine Macrophages

**DOI:** 10.3390/ijms22062893

**Published:** 2021-03-12

**Authors:** Asami Watahiki, Seira Hoshikawa, Mitsuki Chiba, Hiroshi Egusa, Satoshi Fukumoto, Hiroyuki Inuzuka

**Affiliations:** 1 Center for Advanced Stem Cell and Regenerative Research, Tohoku University Graduate School of Dentistry, Sendai 980-8575, Japan; asami.watahiki.c4@tohoku.ac.jp (A.W.); seira.hoshikawa.e5@tohoku.ac.jp (S.H.); mitsuki.chiba.q5@dc.tohoku.ac.jp (M.C.); egu@dent.tohoku.ac.jp (H.E.); 2 Division of Molecular and Regenerative Prosthodontics, Tohoku University Graduate School of Dentistry, Sendai 980-8575, Japan; 3 Division of Pediatric Dentistry, Tohoku University Graduate School of Dentistry, Sendai 980-8575, Japan; 4 Department of Pediatric Dentistry, Graduate School of Dental Science, Kyushu University, Fukuoka 812-8582, Japan

**Keywords:** lipin2, Majeed syndrome, macrophage, autoinflammatory bone disorder, inflammation, osteoclastogenesis

## Abstract

Lipin2 is a phosphatidate phosphatase that plays critical roles in fat homeostasis. Alterations in *Lpin2*, which encodes lipin2, cause the autoinflammatory bone disorder Majeed syndrome. Lipin2 limits lipopolysaccharide (LPS)-induced inflammatory responses in macrophages. However, little is known about the precise molecular mechanisms underlying its anti-inflammatory function. In this study, we attempted to elucidate the molecular link between the loss of lipin2 function and autoinflammatory bone disorder. Using a *Lpin2* knockout murine macrophage cell line, we showed that lipin2 deficiency enhances innate immune responses to LPS stimulation through excessive activation of the NF-κB signaling pathway, partly because of TAK1 signaling upregulation. Lipin2 depletion also enhanced RANKL-mediated osteoclastogenesis and osteoclastic resorption activity accompanied by NFATc1 dephosphorylation and increased nuclear accumulation. These results suggest that lipin2 suppresses the development of autoinflammatory bone disorder by fine-tuning proinflammatory responses and osteoclastogenesis in macrophages. Therefore, this study provides insights into the molecular pathogenesis of monogenic autoinflammatory bone disorders and presents a potential therapeutic intervention.

## 1. Introduction

Alterations in human *Lpin2* cause Majeed syndrome, a rare inherited autosomal recessive autoinflammatory bone disorder that is characterized by early onset chronic multifocal osteomyelitis, neutrophilic skin inflammation, and dyserythropoietic anemia [1,2,3,4,5]. Patients with a monogenic form of this disorder present with poor prognosis; however, IL-1β blockade has showed promising clinical outcomes [6]. *Lpin2* encodes lipin2, a member of the lipin1–3 protein family. Lipins are phosphatidic acid phosphatases (PAPs) that are critical for fine-tuning cellular lipid metabolism [7,8]. The mechanisms by which loss-of-function *Lpin2* mutations contribute to autoinflammatory bone disorder development are largely unknown.

Toll-like receptors (TLRs) are a family of pattern recognition receptors (PRRs) that are critical for proper innate immune responses. Among the 10 TLRs in humans, TLR2/4 are activated by lipopolysaccharides (LPS) to transmit downstream signaling, such as the NF-κB and MAP kinase pathways, to elicit proinflammatory transcription [9,10]. Notably, recent findings link lipin2 function to innate immunity and inflammation and are consistent with the pathophysiological conditions in Majeed syndrome [8,11]. *Lpin2* deficiency in murine and human macrophages augmented the proinflammatory cytokines IL-1β and TNFα [12,13]. Lipin2-deficient macrophages displayed enhanced MAP kinase and P2X7 signaling pathway activation and NLRP3 inflammasome formation in response to LPS and ATP stimulation [12]. *Lpin2* knockout mice exhibited elevated serum IL-1β and TNFα levels and upregulated hepatic and splenic proinflammatory transcripts in response to a high intraperitoneal LPS dose [12]. These data suggest that lipin2 constrains proinflammatory responses in vitro and in vivo. However, the molecular mechanisms by which lipin2 modulates cellular inflammatory signaling have not been clarified.

Monogenic *Lpin2* mutation causes familial chronic multifocal osteomyelitis and osteolytic foci that are characteristic of Majeed syndrome [14,15]. Notably, a very recent study has reported enhanced osteoclastogenesis in monocyte-derived M2-like macrophages from a patient with Majeed syndrome [16]. In this study, we aimed to determine the molecular link between loss of lipin2 function and autoinflammatory bone disorder pathophysiology. Our findings may advance the understanding of the molecular pathogenesis and therapeutic intervention for *Lpin2*-mutation and other monogenic autoinflammatory bone disorders.

## 2. Results

### 2.1. Lipin2 Deficiency Enhances Proinflammatory Responses in Macrophages by Modulating Inflammatory Transcription

**Figure 1:**
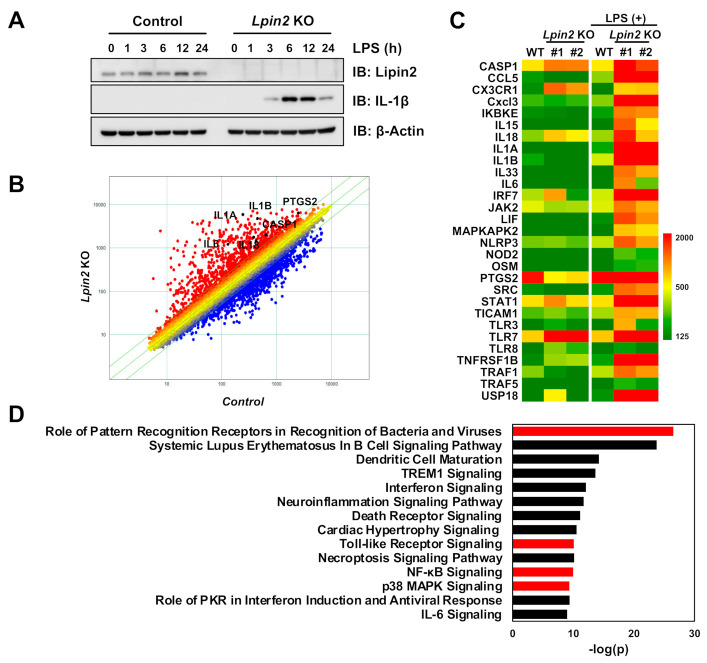
*Lpin2* knockout elicits proinflammatory gene signature in RAW-D macrophages. (**A**) Immunoblot (IB) analysis of whole-cell lysates (WCLs) derived from control and *Lpin2* knockout (KO) RAW-D cells treated with 200 ng/mL lipopolysaccharide (LPS). The cells were harvested for the IB analysis at the indicated time points. Wild-type (WT) cells were used as control. (**B**) Scatterplot comparing gene expression profiles of control (WT) (x-axis) and *Lpin2* KO (y-axis) RAW-D cells treated with 200 ng/mL LPS for 4 h (red, upregulation; blue, downregulation; fold change > 2.0). (**C**) Heatmap of transcription levels of selected inflammation-related genes in control and *Lpin2* KO RAW-D cells (clone #1 and #2) treated with 200 ng/mL LPS for 4 h. (**D**) “Canonical Pathways” analyzed by Qiagen ingenuity pathway analysis software for differentially expressed genes (DEGs) in *Lpin2* KO RAW-D cells (fold change > 2.0) treated with 200 ng/mL LPS for 4 h.

To determine the physiological role of lipin2 in regulating macrophage proinflammatory and osteoclastic signaling, we knocked out *Lpin2* in murine macrophage RAW-D cells, a RAW264.7 subclone with a higher potential to differentiate into osteoclasts [17,18]. We treated the control and the *Lpin2* knockout cells with LPS and compared their responsiveness to proinflammatory stimulation. As shown in a previous study [12], marked IL-1β protein induction was observed in the *Lpin2* knockout cells following LPS stimulation (Figure 1A). We then investigated the role of lipin2 in LPS-elicited proinflammatory transcription. We conducted a microarray expression analysis using LPS-treated or untreated *Lpin2* knockout and control RAW-D cells. Scatterplots and heatmaps showed upregulated proinflammatory transcripts in response to LPS in *Lpin2* knockout cells (Figure 1B,C). Qiagen ingenuity pathway analysis (IPA) of differentially expressed genes (DEGs) (fold change > 2) in *Lpin2* knockout cells identified PRRs, TLRs, NF-κB, and p38 MAP kinase signaling (Figure 1D). These data indicate that lipin2 deficiency promotes macrophage proinflammatory responses.

### 2.2. Lpin2 Knockout Enhances the NF-κB Signaling Pathway in RAW-D Cells

**Figure 2:**
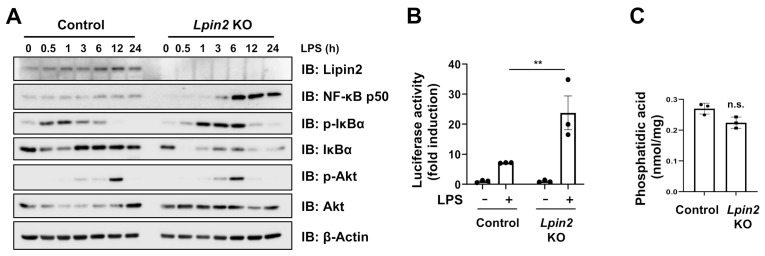
*Lpin2* knockout induces NF-κB signaling in RAW-D macrophages. (**A**) IB analysis of WCLs derived from control and *Lpin2* KO RAW-D cells treated with 200 ng/mL LPS. The cells were harvested for IB analysis at the indicated time points. (**B**) A luciferase reporter assay showing relative NF-κB activity in control and *Lpin2* KO RAW-D cells with or without 200 ng/mL LPS treatment for 4 h. Data are means ± SD (*n* = 3). ** *P* < 0.01; not significant, One-way ANOVA followed by multiple comparisons test. (**C**) Quantification of intracellular phosphatidic acid (PA) in control (WT) and *Lpin2* KO RAW-D cells. Data are means ± SD (*n* = 3), n.s., not significant, Student’s *t*-test.

To confirm whether lipin2 modulates inflammatory responses via TLR signaling, we assessed the activation status of the TLR4 downstream inflammatory signaling. We observed that *Lpin2* knockout cells showed IκB phosphorylation and downregulation, and accelerated p50 processing, which are NF-κB activation read-outs, while no impact on Akt pathway was observed (Figure 2A). A luciferase reporter assay validated the LPS-mediated NF-κB overactivation in *Lpin2* knockout cells (Figure 2B). We investigated whether the NF-κB signaling pathway was altered in response to accumulation of phosphatidic acid (PA), a lipin2 PAP substrate functioning as a signaling molecule [19]. However, the gross PA level did not markedly differ between *Lpin2* knockout and control cells (Figure 2C).

### 2.3. Elevated IRAK Signaling Induces Downstream Pathways in Lpin2 Knockout Macrophages

**Figure 3:**
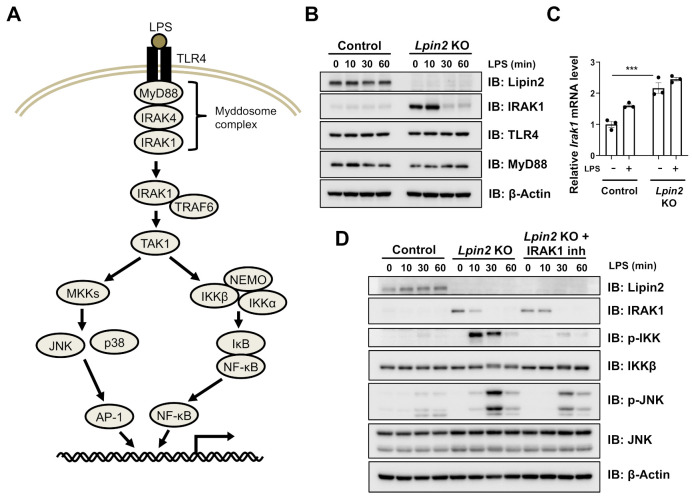
Elevated IRAK signaling induces downstream pathways in *Lpin2* knockout RAW-D cells. (**A**) A scheme of the TLR4 signaling pathway. (**B**) IB analysis of WCLs derived from control (WT) and *Lpin2* KO RAW-D cells treated with 200 ng/mL LPS. The cells were harvested for IB analysis at the indicated time points. (**C**) RT-PCR was performed to determine relative *Irak1* mRNA expression in control and *Lpin2* KO RAW-D cells treated with 200 ng/mL LPS for 4 h. *Gapdh* was used for normalization. Data are means ± SEM (*n* = 3). *** *P* < 0.001, One-way ANOVA followed by multiple comparison test. (**D**) IB analysis of WCLs derived from control and *Lpin2* KO RAW-D cells treated with 200 ng/mL LPS. Cells were treated with either DMSO or 10 µM IRAK inhibitor IRAK1/4 as indicated for 1 h before LPS treatment. The cells were harvested for IB analysis at the indicated time points.

Our IPA and western blotting results demonstrated that lipin2 is vital to the TLR4 downstream signaling pathways (Figure 1 andFigure 2). To explore the underlying mechanism of lipin2 deficiency-induced proinflammatory signaling overactivation, we evaluated TLR-proximal adaptor protein and kinase, MyD88, and IRAK1, which comprise the Myddosome, a signaling complex formed in response to TLR activation [20,21] (Figure 3A). IRAK1 was remarkably upregulated, whereas the levels of TLR4 and MyD88 were unchanged in *Lpin2* knockout cells stimulated by LPS within a short period of 60 min (Figure 3B). IRAK1 accumulation might be regulated by pre- and post-transcriptional control because the induction of the protein level in *Lpin2* knockout cells is greater when compared to two-fold induction of transcription level (Figure 3C). IRAK1 transmits TLR4 signaling downstream by complexing with IRAK4 [22,23]. To test whether the IRAK complex activity results in the overactivation of downstream NF-κB signaling in *Lpin2* knockout cells, we treated *Lpin2* knockout cells with the IRAK inhibitor, IRAK1/4. The inhibitor treatment suppressed the activation of the downstream NF-κB signaling pathway as well as JNK signaling, the activity of which is reported to increase in lipin2-deficient macrophages [12,13] (Figure 3D).

### 2.4. Elevated TAK1 Activity Induces Downstream Signaling in Lpin2 Knockout Macrophages

**Figure 4:**
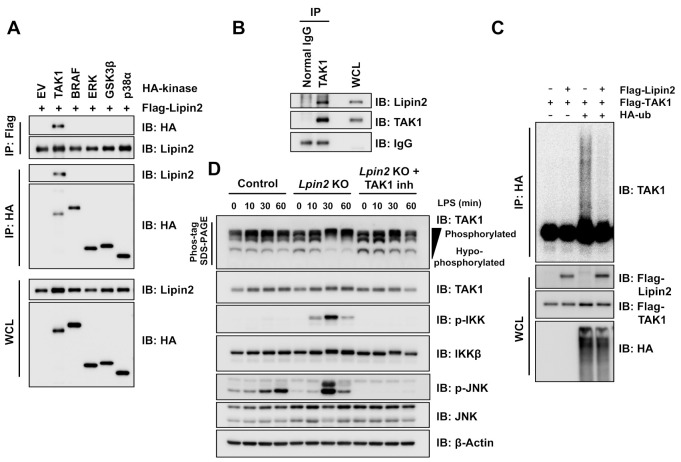
Elevated TAK1 activity induces downstream pathways in *Lpin2* knockout RAW-D cells. (**A**) IB analysis of WCLs and anti-Flag/anti-HA IPs derived from 293T cells transfected with the indicated expression plasmids. At 48 h after transfection, the cells were harvested for IP. (**B**) IB analysis of WCLs and anti-TAK1 IPs derived from RAW-D cells. Normal mouse IgG was used as control IP. (**C**) In-cell TAK1 ubiquitination assay. IB analysis of WCLs and anti-HA IPs derived from 293T cells transfected with the indicated expression plasmids. At 36 h after transfection, the cells were pretreated with 15 µM MG132 proteasome inhibitor for 12 h before harvesting. (**D**) IB analysis of WCLs derived from control (WT) and *Lpin2* KO RAW-D cells treated with 200 ng/mL LPS. Cells were pretreated with DMSO or 1 µM 5Z-7-oxozeaenol TAK1 inhibitor as indicated for 1 h before LPS treatment. The cells were harvested for IB analysis at the indicated time points. For enhanced electrophoretic separation of phosphorylated TAK1 species, the WCLs were resolved by Phos-tag SDS-PAGE as indicated.

It was reported that the founding family member lipin1 serves as a scaffold for several cytoplasmic and nuclear signaling molecules to regulate lipid and energy metabolism [24,25,26,27,28,29]. We hypothesized that lipin2 also has a similar ability for proinflammatory signaling control in macrophages. We screened lipin2 interacting proteins by Flag-lipin2 co-immunoprecipitation (IP)/mass spectrometry and identified TAK1 as a potential lipin2 interacting protein (data not shown). The TAK1–TAB complex transduces proinflammatory signaling by activating NF-κB and MAP kinase signaling via IKKβ and MAPKK phosphorylation [30,31] (Figure 3A). We speculated that this signaling complex might be a potential lipin2 target. To confirm the interaction between lipin2 and TAK1, we ectopically co-expressed different HA-tagged protein kinases and Flag-lipin2 in 293T cells and performed a co-IP assay. Lipin2 only interacted with TAK1 (Figure 4A). We also demonstrated endogenous interaction between TAK1 and lipin2 (Figure 4B). TAK1 activity is positively controlled by phosphorylation and K63-linked polyubiquitination [32,33]. Thus, we examined whether lipin2 interaction affects TAK1 polyubiquitination. Lipin2 co-expression markedly impaired TAK1 polyubiquitination (Figure 4C). We observed the phosphorylated active form of TAK1 in LPS-treated *Lpin2* knockout cells (Figure 4D). TAK1 inhibitor treatment suppressed NF-κB signaling as well as JNK activation (Figure 4D).

### 2.5. Lipin2 Deficiency Promotes RANKL-Dependent Osteoclastogenesis and Osteoclastic Resorption Activity in RAW-D Macrophages

**Figure 5:**
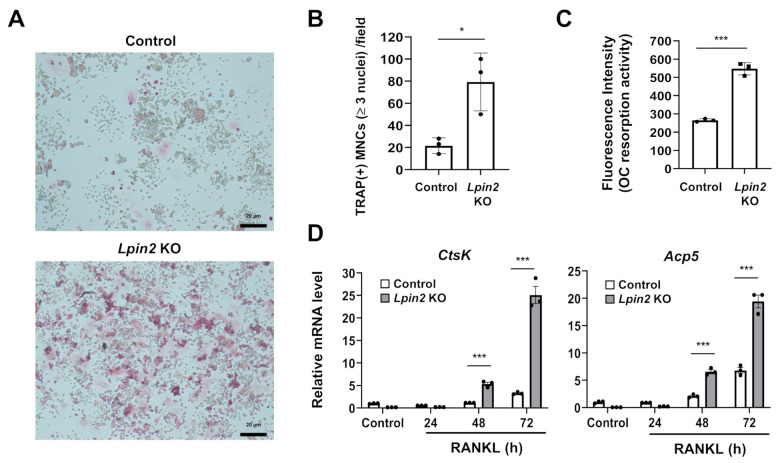
Lipin2 deficiency promotes RANKL-mediated osteoclast formation in RAW-D cells. (**A**) Tartrate-resistant acid phosphatase (TRAP) staining of multinuclear cells (MNCs). Control (WT) and *Lpin2* KO RAW-D cells were cultured in the presence of 50 ng/mL RANKL for 3 d and fixed for staining. Scale bar, 20 μm. Magnification, 50×. (**B**) TRAP-positive MNCs with ≥3 nuclei were counted for each group. Data are means ± SD (*n* = 3). * *P* < 0.05, Student’s *t*-test. (**C**) Quantification of osteoclastic (OC) resorption activity. Control and *Lpin2* KO RAW-D cells were cultured in fluoresceinated calcium phosphate-coated plates in the presence of 100 ng/mL RANKL for 3 d. OC resorption activity was evaluated by measuring fluorescence intensity in conditioned medium. Data are means ± SD (*n* = 3). *** *P* < 0.001, Student’s *t*-test. (**D**) RT-PCR was performed to determine relative mRNA expression levels of the osteoclastic marker genes *Ctsk* and *Acp5* in control and *Lpin2* KO RAW-D cells treated with 50 ng/mL RANKL. The cells were harvested for RNA extraction at the indicated time points. *Gapdh* was used for normalization. Data are means ± SEM (*n* = 3). *** *P* < 0.001. One-way ANOVA followed by the multiple comparisons test.

*Lpin2* alterations cause multifocal osteomyelitis, and there might be a major link between lipin2 function and the intrinsic osteolytic pathophysiology. Thus, we explored the possibility that lipin2 deficiency promotes osteoclastogenesis in macrophages. *Lpin2* knockout cells displayed higher potential than control cells for RANKL-mediated osteoclast-like multinuclear cell (MNC) formation (Figure 5A,B). *Lpin2* knockout cells showed increased osteoclastic resorption activity (Figure 5C). The transcription levels of the osteoclast markers, *Ctsk* and *Acp5*, were markedly increased in *Lpin2* knockout cells (Figure 5D). These data demonstrate that lipin2 deficiency increases RANKL-dependent formation of osteoclast-like MNCs.

### 2.6. Lipin2 Negatively Regulates NFATc1 Activity

**Figure 6:**
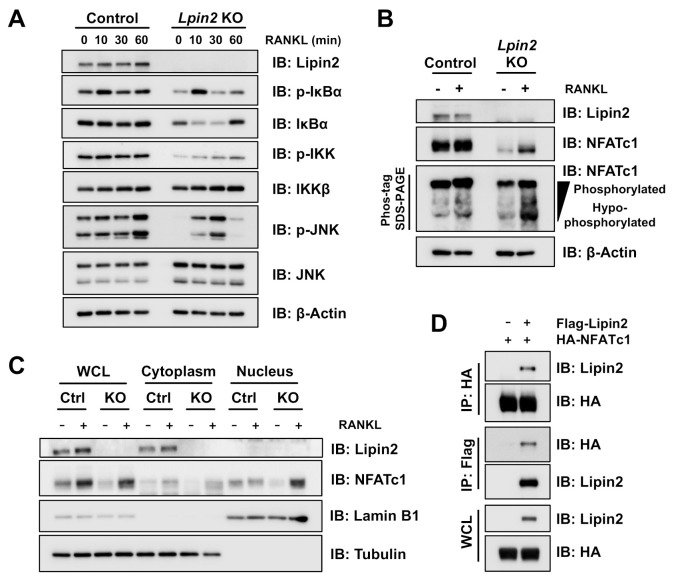
Lipin2 negatively regulates NFATc1 nuclear translocation in RAW-D cells. (**A**) IB analysis of WCLs derived from control (WT) and *Lpin2* KO RAW-D cells treated with 50 ng/mL RANKL. The cells were harvested for IB analysis at the indicated time points. (**B**) IB analysis of WCLs derived from control and *Lpin2* KO RAW-D cells treated with 50 ng/mL RANKL for 24 h. The cells were harvested for IB analysis at the indicated time points. For enhanced electrophoretic separation of phosphorylated NFATc1 species, the WCLs were resolved by Phos-tag SDS-PAGE as indicated. (**C**) IB analysis of WCL, cytoplasmic, and nuclear fractions derived from control (Ctrl) and *Lpin2* KO RAW-D cells treated with 50 ng/mL RANKL for 24 h. Tubulin and Lamin B1 are shown as markers of cytoplasm and nucleus, respectively. (**D**) IB analysis of WCLs and anti-HA/anti-Flag IPs derived from 293T cells transfected with the indicated expression plasmids.

Since MAP kinase and NF-κB signaling play critical roles in osteoclast differentiation [34], we conducted western blot analysis to evaluate their activation status. RANKL stimulation unexpectedly did not induce excessive MAP kinase and NF-κB pathway activation in *Lpin2* knockout cells (Figure 6A). However, we observed impaired NFATc1 phosphorylation in *Lpin2* knockout cells (Figure 6B). NFATc1 is a master transcription regulator of osteoclastogenesis. RANKL stimulation is known to promote calcineurin-mediated NFATc1 dephosphorylation, inducing its nuclear translocation and transactivation activity [35,36,37]. We observed an enhanced RANKL-induced NFATc1 accumulation in the nucleus of *Lpin2* knockout cells (Figure 6C). A previous study demonstrated the physical interaction between lipin1 and NFATc4, which resulted in the suppression of NFATc4 transcriptional activity and downstream proinflammatory cytokine expression [28]. For this reason, we tested whether lipin2 interacts with NFATc1. A co-IP assay with transfected 293T cells demonstrated that lipin2 binds to NFATc1 (Figure 6D).

## 3. Discussion

*Lpin2* alterations cause Majeed syndrome, which is characterized by chronic autoinflammatory multifocal osteomyelitis [3,4,5,6]. Therefore, lipin2 could prevent excessive proinflammatory signal activation. A previous study demonstrated that lipin2 plays a crucial role in limiting p38, ERK, and JNK activities in inflammatory macrophages. Overactivation of these kinases by lipin2 deficiency facilitates excessive *Il1b* mRNA expression and mature IL-1β overproduction [12]. In the present study, we showed that *Lpin2* depletion in a murine macrophage cell line resulted in an elevated NF-κB signaling pathway. Therefore, lipin2 may modulate the TLR4 downstream inflammatory signaling axes, MAP kinase, and NF-κB pathways.

Besides PAP function, lipin1 acts as a scaffold for various signaling molecules, such as PPARα, NFATc4, SREBP1, and ERK1/2 [24,25,27,28]. Lipin2, like lipin1, interacts with PPARα and modifies its transcriptional activity [38]. These foregoing studies suggest that lipin family members are multifunctional in nature. In this study, we demonstrated that lipin2 negatively modulates TAK1 activity. We also found that IRAK1 is upregulated in *Lpin2* knockout cells. Since IRAK1 is an unstable protein subjected to proteasomal degradation [22,39], lipin2 may possibly contribute to this process. However, further investigations are required to elucidate the precise molecular mechanisms through which lipin2 controls the related signaling pathways. In summary, our findings suggest that lipin2 may play a critical role in suppressing NF-κB signaling in part through the TAK1 pathway. Targeting the overactivated signaling might be an efficient therapeutic strategy, and the use of specific inhibitors should be explored for their effectiveness in the treatment of lipin2-deficient autoinflammatory disorder.

A recent study has identified that the disease-associated *Lpin2* mutation is linked to elevated osteoclast formation. M2-like macrophages derived from the patient with Majeed syndrome display enhanced osteoclastogenesis. The macrophages show a proinflammatory phenotype with elevated NFATc1 and phosphorylated JNK levels [16]. Here, we revealed that *Lpin2* knockout in RAW-D macrophages promotes osteoclast-like MNC formation by activating the NFATc1 pathway. These findings may provide evidence of the involvement of lipin2 in bone metabolism. However, one limitation is that *Lpin2* knockout mice lack phenotypes resembling osteomyelitis. Hence, in-depth studies may be necessary to bridge the gap between human pathophysiology and the absence of osteomyelitis phenotype in mice [8]. In this work, we presented a model demonstrating that lipin2 regulates proinflammatory and osteoclastic signaling via upstream modulation of NF-κB and NFATc1 signaling. This research may lay the foundation for elucidating the pathogenesis of monogenic autoinflammatory bone disorders, which may help in the development of novel therapeutic strategies.

## 4. Materials and Methods

### 4.1. Cell Culture

Murine macrophage RAW-D cells are subclones of RAW264.7 and were kindly gifted by Dr. Toshio Kukita [17,18]. The 293T (RRID:CVCL_0063) and HeLa (RRID:CVCL_0030) cells were obtained from the American Type Culture Collection (ATCC; Manassas, VA, USA). The RAW-D cells were cultured in α-MEM (modified Eagle’s medium). The 293T and HeLa cells were cultured in DMEM (Dulbecco’s modified Eagle’s medium). Each medium was supplemented with 10% (*v/v*) fetal bovine serum (FBS), 100 U penicillin, and 100 μg/mL streptomycin. Transfection was performed with polyethylenimine (PEI) [40]. *Lpin2* knockout cells were generated using a previously described method [41]. Briefly, RAW-D cells were transfected with mouse Lipin-2 CRISPR/Cas9 KO and HDR plasmids (Santa Cruz Biotechnology, Dallas, TX, USA). At 48 h after transfection, the cells were selected with 1.5 μg/mL puromycin for 3 d and reseeded for clonal isolation on a 96-well plate. Lipopolysaccharides (LPS-EB: standard LPS from *E. coli* 0111) were purchased from Nacalai Tesque (Kyoto, Japan). Recombinant human sRANKL was procured from Fujifilm Wako (Osaka, Japan). IRAK1/4 (IRAK inhibitor) and 5Z-7-oxozeaenol (TAK1 inhibitor) were obtained from Merck (Darmstadt, Germany).

### 4.2. Antibodies and Plasmids

The anti-Lipin-2 antibody (A303-703A) was purchased from Bethyl Laboratories (Montgomery, TX, USA). Anti-IL-1β (3A6) (12242), anti-phospho-IκBα (S32/36) (5A5) (9246), anti-IκBα (44D4) (4812), anti-Phospho-SAPK/JNK (Thr183/Tyr185) (81E11) (4668), anti-JNK2 (56G8) (9258), Anti-phospho-Akt (ser473) (D9E) (4060), anti-Akt (pan) (40D4) (2920), anti-IRAK1 (D51G7) (4504), anti-MyD88 (D80F5) (4283), anti-TAK1 (D94D7) (4505), anti-phospho-IKKα/β (Ser176/180) (16A6) (2697), anti-IKKβ (D30C6) (8943), and anti-Myc-Tag (71D10) (2278) antibodies were purchased from Cell Signaling Technology (Danvers, MA, USA). Anti-HA (Y-11) (sc-805), anti-NFATc1 (7A6) (sc-7294), anti-Lamin B1 (S-20) (sc-30264), anti-α-Tubulin (TU-02) (sc-8035), and anti-β-Actin (C4) (sc-47778), anti-NF-κB p50 (sc-8414), anti-TLR4 (sc-293072) antibodies, and anti-Tak1 (C-9) antibody agarose conjugated (sc-7967 AC) were purchased from Santa Cruz Biotechnology. Anti-Flag monoclonal (018-22381) antibodies were purchased from Fujifilm Wako. Flag-TAK1 and HA-NFATc1 expression plasmids were constructed by subcloning the appropriate PCR fragments into pcDNA3-Flag and pcDNA3-HA, respectively. HA-BRAF, HA-ERK, and HA-p38 expression plasmids were kindly provided by Dr. Wenyi Wei. pRK6-HA-TAK1 (14753), pRK5-HA-ubiquitin (HA-ub) (17608), and pcDNA-HA-GSK3β (14753) were purchased from Addgene (Watertown, MA, USA). pcDNA-Flag-mouse lipin-2 was a kind gift from Dr. Seung-Hoi Koo. 

### 4.3. Immunoblots and Immunoprecipitation

The cells were lysed in an NP-40 cell lysis buffer (50 mM Tris-Cl [pH 7.5], 120 mM NaCl, and 0.5% (*v/v*) NP-40) supplemented with a cOmplete protease inhibitor cocktail (Sigma-Aldrich Corp., St. Louis, MO, USA) and phosphatase inhibitors (PhosSTOP; Roche Diagnostics, Mannheim, Germany). The lysate protein concentrations were measured with Bio-Rad protein assay dye (Bio-Rad Laboratories, Hercules, CA, USA). Forty micrograms of whole-cell lysate was dissolved in an SDS sample buffer and resolved by SDS-PAGE. For Phos-tag SDS-PAGE, each sample was resolved in an SDS-PAGE gel containing 50 μM Phos-tag (Fujifilm Wako) according to the manufacturer’s instructions. The gels were transferred to polyvinylidene difluoride (PVDF) membranes (Bio-Rad Laboratories), which were then blocked with 5% (*w/v*) nonfat dry milk in Tris-buffered saline with 0.05% (*v/v*) Tween 20 (TBST; pH 8.0) and probed with primary antibodies, as indicated in the figures (1:1000–1:4000 with 5% (*w/v*) nonfat dry milk in TBST). For immunoprecipitation, the cells were harvested in an NP-40 cell lysis buffer containing protease and phosphatase inhibitors. Six hundred microliters (corresponding to 1 mg of cell lysate) were incubated with 8 μL of anti-HA or Flag-tag antibody-conjugated bead slurry or 20 μL of anti-TAK1 antibody-conjugated bead slurry at 4 °C, with gentle rocking for 4 h. The beads were washed 5× with 1 mL of an NP-40 washing buffer (20 mM Tris [pH 8.0], 100 mM NaCl, 1 mM EDTA, and 0.5% (*v/v*) NP-40), resuspended in 50 μL of 2×SDS sample buffer, and heated at 90 °C for 5 min. Each 15 μL of eluate and 60 μg of whole-cell lysate were separated by SDS-PAGE followed by immunoblot analysis with primary antibodies, as indicated and TrueBlot IgG HRP secondary antibodies (Rockland Immunochemicals, Limerick, PA, USA).

### 4.4. RT-PCR and Microarray Expression Analyses

Total RNA was extracted with TRIzol reagent (Thermo Fisher Scientific, Waltham, MA, USA). The reverse transcription (RT) reaction was performed with ReverTra Ace qPCR RT Master Mix (TOYOBO, Osaka, Japan). RT-PCR was performed with SYBR Select Master Mix (Thermo Fisher Scientific). Relative gene expression was calculated by the 2^−ΔΔCt^ method [42]. *Gapdh* normalized the transcript levels. All procedures were performed according to the manufacturers’ instructions. The primers used for the PCR reactions are as follows: mouse *Irak1*, Forward, 5′-CAGAACCACCACAGATCATCATC-3′, Reverse, 5′-AGGCTTCAATTCCAATAGCATCA-3′; mouse *Ctsk*, Forward, 5′-GAAGAAGACTCACCAGAAGCAG-3′, Reverse, 5′-TCCAGGTTATGGGCAGAGATT-3′; mouse *Acp5*, Forward, 5′-CACTCCCACCCTGAGATTTGT-3′, Reverse, 5′-CATCGTCTGCACGGTTCTG-3′; mouse *Gapdh*, Forward, 5′-GGAGCGAGATCCCTCCAAAAT-3′, Reverse, 5′-AGTGATGGCATGGACTGTGGT-3′. For the DNA microarray analysis, total RNA was extracted with the RNeasy mini kit and QIAshredder (Qiagen, Hilden, Germany). RNA quality and microarray expression analyses were conducted by Riken Genesis (Kawasaki, Japan) using mouse Clariom^TM^ S arrays (Affymetrix, Santa Clara, CA, USA). Data were analyzed by Tohoku Kagaku (Sendai, Japan). All microarray data were deposited in the NCBI Gene Expression Omnibus (GEO) database (GSE166741). Pathway analysis was carried out using the ingenuity pathway analysis (IPA) tool (Qiagen).

### 4.5. Osteoclast Formation and Osteoclastic Resorption Activity Assays

Murine macrophage RAW-D cells were cultured with 50 ng/mL RANKL for 3 d to induce osteoclastogenesis. The cells were fixed with 3.7% (*v/v*) formaldehyde, and osteoclast formation was detected by tartrate-resistant acid phosphatase (TRAP) staining. Images were acquired using Vert A1 microscopy and ZEN software (ZEISS, Jena, Germany). TRAP-positive MNCs containing ≥3 nuclei were scored as differentiated osteoclast-like cells. Osteoclastic resorption activity measurement was conducted with a bone resorption assay kit (PG Research, Kodaira, Japan) according to the manufacturer’s instructions. Briefly, RAW-D cells were seeded at 1 × 10^4^/well on a 48-well plate coated with fluoresceinated calcium phosphate and cultured for 3 d in phenol red-free medium containing 100 ng/mL RANKL. Osteoclastic resorption activity was evaluated by measuring the fluorescence intensity of 100 μL of conditioned medium at excitation/emission (Ex/Em) wavelengths of 485/535 nm in a SpectraMax M2e microplate reader (Molecular Devices LLC, San Jose, CA, USA).

### 4.6. Cellular Fractionation

Cells were fractioned according to a previously described protocol [43]. Briefly, 10^7^ RAW-D cells were collected in 1 mL of ice-cold phosphate-buffered saline (PBS), centrifuged at 1000× *g* for 3 min at 4 °C, and the cell pellets were resuspended in 900 μL of ice-cold PBS containing 0.1% (*v/v*) NP-40 plus protease inhibitor. Afterwards, 300 μL of whole-cell lysate fraction was withdrawn, and the remainder was centrifuged at 10,000× *g* for 10 s at 4 °C. After that, 400 μL of supernatant was collected and centrifuged at 10,000× *g* for 10 s at 4 °C, and 200 μL of supernatant (cytosolic fraction) was collected. The pellets (nuclear fraction) obtained after the first spin were washed twice with 1 mL of ice-cold 0.1% (*v/v*) NP40/PBS and dissolved in a 1×SDS sample buffer. The nuclear and whole-cell lysate fractions dissolved in the SDS sample buffer were sonicated with a VP-050 microprobe (TAITEC, Koshigaya, Japan).

### 4.7. Phosphatidic Acid (PA) Measurement

The PA content was measured with a PicoProbe phosphatidic acid assay kit (BioVision, Milpitas, CA, USA) according to the manufacturer’s instructions. Briefly, 10^6^ WT and 10^6^ *Lpin2* knockout cells were harvested and lysed with 1 mL of a PA assay buffer, and their protein concentrations were determined with Bio-Rad protein assay dye. The lipids were extracted by adding 3.75 mL of chloroform/methanol/12 N HCl (2:4:0.1), 1.25 mL of chloroform, and 1.25 mL of 1 M NaCl, vortexing, centrifuging at 3000× *g* for 10 min at RT, and collecting the organic layer. The chloroform was evaporated in the hood for overnight, and the PA was dissolved in 5% (*w/v*) Triton X-100, converted to a fluoresceinated PA intermediate, and quantified by measuring the latter at Ex/Em wavelengths of 535 nm/587 nm in a SpectraMax M2e microplate reader (Molecular Devices LLC).

### 4.8. Luciferase Reporter Assay

Luciferase assay was carried out using the Dual-Glo Luciferase Assay System (Promega, Madison, WI, USA) according to the manufacturer’s instructions. Briefly, control or *Lpin2* knockout RAW-D cells were transfected with pGL4.32[Luc2P/NF-kB-RE] firefly luciferase reporter and pRL-TK renilla luciferase expression plasmids (Promega) at a ratio of 50:1. At 24 h after transfection, cells were stimulated with 200 ng/mL LPS. After 4 h, cells were harvested for evaluating reporter activities. The firefly and renilla luciferase activities were measured in a multimode reader, TriStar2 LB942 (Berthold Technologies, Bad Wildbad, Germany). The values of firefly luciferase activities were normalized with that of renilla luciferase.

### 4.9. Mass Spectrometry

HeLa cells were transfected with pcDNA3-Flag-lipin2. After 36 h, the cells were treated with 10 μM MG132 for 12 h and harvested in an NP-40 cell lysis buffer containing protease and phosphatase inhibitors. Lysate from empty vector-transfected cells was prepared and used as the control IP sample. Five hundred microliters corresponding to 0.5 mg of cell lysate was incubated with 10 μL of anti-Flag antibody-conjugated bead slurry (Sigma-Aldrich Corp) at 4 °C, with gentle rocking for 2 h. The beads were washed twice with 1 mL of an NP-40 washing buffer and twice with TBST. Samples were purified by liquid chromatography/tandem mass spectrometry (LC-MS/MS) as previously described [44] at Kazusa Genome Technologies in the Kazusa DNA Research Institute (Kisarazu, Japan).

### 4.10. Quantification and Statistical Analysis

Data are means ± SD or SEM for ≥3 independent experiments or biological replicates. Between-group differences were analyzed by one-way ANOVA with multiple comparison tests or Student’s *t*-test. Statistical analyses were performed in GraphPad Prism9 (GraphPad Software, San Diego, CA, USA). *P* < 0.05 was considered a statistically significant difference.

## Data Availability

Microarray data was deposited in the GEO database (GSE166741).
